# Determination of autoantibodies in dogs with diabetes mellitus

**DOI:** 10.14202/vetworld.2021.2694-2698

**Published:** 2021-10-22

**Authors:** Franco González-Villar, Francisco Pérez-Bravo

**Affiliations:** 1 Doctoral Program in Silvoagropecuary and Veterinary Sciences, University of Chile, South Campus, 11315 Santa Rosa, La Pintana, Santiago, Chile; 2 Department of Nutrition, Institute of Nutrition and Food Technology, University of Chile, Santiago, Chile.

**Keywords:** autoantibodies, autoimmunity, diabetes, dogs

## Abstract

**Background and Aim:**

The classification of diabetes mellitus (DM) in dogs has been controversial as currently canine insulin-dependent DM is classified together with absolute insulin deficiency, non-insulin-dependent DM, and relative insulin deficiency. Studies on human autoantibodies evaluated in canines with DM, such as anti-glutamic acid decarboxylase (GAD65), anti-islet antigen 2 (IA2), and anti-zinc transporter isoform 8 (ZnT8), have been inconclusive. Thus, this study was designed to establish the serological profile of anti-GAD65, anti-IA2, and anti-ZnT8 antibodies in a group of dogs with and without DM.

**Materials and Methods:**

Sixty-one dogs, including 31 patients with DM (with and without insulin treatment) and 30 patients without DM (normal weight and obese), were included for determining autoantibodies using a human enzyme-linked immunosorbent assay (ELISA) detection system for type 1 DM.

**Results:**

This study found the presence of anti-IA2 antibodies in 58% of the sample (18/31 patients with DM); however, the presence of anti-GAD65 was not detected, and anti-ZnT8 was found in 3 (9.6%) patients with DM.

**Conclusion:**

This study showed a higher positive frequency of anti-IA2 antibodies in a sample of canine with DM, indicating that alterations in the signaling vesicle tyrosine phosphatase 2 lead to lower insulin release and thus to an increase in patients’ glycemia. These preliminary results should be taken with caution and corroborated by a canine-specific assay when an ELISA is available for such determination.

## Introduction

Diabetes mellitus (DM) is a metabolic disease with the participation of genetics and some environmental components. The global increase in the incidence of DM may be partially or fully due to the lack of the ability to produce insulin or a loss of insulin sensitivity in peripheral tissues [1].

The islets of Langerhans in humans and canines are different. In humans, 54% of these cells correspond to β-cells, 35% correspond to α-cells, and 11% correspond to other cells, whereas, in canines, 80% of the islets correspond to β-cells, 10% correspond to α-cells, and 10% correspond to pancreatic polypeptide and somatostatin. The loss of β-cells may explain numerous insulin-dependent canines by drawing a parallel between humans and canines with type 1 DM [2]. Studies analyzing canine patients with DM have found the presence of polymorphisms in the major histocompatibility complex type 2, specifically for the dog leukocyte antigen (DLA) haplotype. These genes encode glycoproteins with an immunoglobulin structure, consisting of an α-chain and a β-chain transmembrane, which act mainly on antigen-presenting cells and TCD4+ lymphocytes [3]. Studies on canines have found a correlation between the presence of DM in canines and the following DLA haplotypes: DLA-DRB1, DLA-DQA1, and DLA-DQB1 (DRB1 009/DQA1 001/DQB1 008, DRB1 015/DQA1 0061/DQB1 023, and DRB1 002/DQA1 009/DQB1 001), with a significant frequency of these haplotypes in canine patients with DM [4].

The study on autoantibodies in DM has been controversial until today and has not allowed the classification of DM as canine autoimmune type 1 DM. Some authors in the UK have established a different classification between veterinary and human medicine and have classified them as insulin resistant or insulin-deficient with partial deficiency of the pancreatic β function. The classification of a canine DM phenotype would be similar to that of humans with type 1 DM, as it presents an apparent lack of insulin-resistant factors associated with obesity, worsening the state of impaired glucose tolerance. Catchpole *et al*. [5] have cloned and expressed the canine recombinant full-length anti-glutamic acid decarboxylase (GAD65) and the C-terminal region of anti-islet antigen 2 (IA2), demonstrating the presence of autoantibodies in a population of dogs newly diagnosed with DM.

The autoantibodies analyzed in various canine studies have been extrapolated from autoimmunity studies in humans. The autoantibodies include those corresponding to the isoform of GAD65 and those against tyrosine phosphatase 2 (IA-2), which are essential proteins in human pancreatic β-cells and whose functions are to convert glutamate into gamma-aminobutyric acid that acts as a neurotransmitter in the pancreatic β-cell in the case of GAD65. IA-2 is a transmembrane protein expressed in β-cell secretory granules and neuroendocrine cells to generate signaling for insulin formation and excretion [6,7]. The third group of autoantibodies related to the autoimmune component in type 1 DM is anti-insulin antibodies (IAAs). These autoantibodies were evaluated in a canine study where they were found in five of 15 canine patients with DM and negative for anti-GAD65 and anti-IA-2 [8]. The results of studies on anti-GAD65 have been conflicting mainly due to the small number of patients examined [7,9] who demonstrated the presence of 100% positivity for anti-GAD65 in a group of eight canine patients with DM tested using radioimmunoassay. Studies involving a larger sample size of 140 patients have shown the absence of anti-GAD 65 antibodies in canines with DM, which is in contrast to previous findings described [9,10].

Among the latest antibodies investigated in human medicine with an impact on DM are the ones against zinc transporter isoform 8 (ZnT8), a molecule that transports zinc from the cytoplasm into the insulin secretion granule, thus allowing the hexameric conformation of insulin and its subsequent excretion. In humans, improved prediction of DM has been achieved from 90%, with the measurement of anti-GAD 65, anti-IA-2, and anti-IAA autoantibodies, to 96% with the addition of anti-ZnT8 antibody [11]. This transporter protein has been found in the pancreas of canines, presenting the same genomic organization between humans and canines, as they share 98% of conserved amino acids and 70% of residues [12]. To date, only one study has been conducted so far on this protein involving 15 canine patients with DM, where no positive titers for anti-ZnT8 have been detected [8].

In humans with early onset of DM, the presence of islet antibodies has been found in obese patients, indicating an autoimmune state in some patients together with insulin resistance [13]. Moreover, patients on insulin treatment may generate antibodies against endogenous insulin, which increases the systemic inflammatory state, even leading to immune complex microangiopathies, although this is still under discussion [14].

This study was designed to evaluate the presence of autoantibodies against β-cells in canine patients with DM using a human detection system and to establish the relative frequency of these autoantibodies in dogs with DM.

## Materials and Methods

### Ethical approval

The Institutional Review Board of University of Chile approved the procedures of animal care and use for the study.

### Study period and location

The study period was from March 2016 to December 2020. The study was carried out at the Institute of Nutrition and Food Technology, Hospital Medivet, and the Clinical Hospital of the University of Chile.

### Animals

A cohort of 61 patients was studied, distributed as 15 dogs with DM without treatment, 16 dogs with DM with insulin treatment, 15 healthy weight normoglycemic dogs, and 15 obese normoglycemic dogs. The age of the canines included in this study ranged between 1 and 15 years without differentiation by sex or breed: 30 mongrels, 10 Poodles, 4 Beagles, 4 Labrador Retrievers, 4 Samoyeds, 3 Golden Retrievers, and 2 Cocker Spaniel along with one each of Fox Terrier, Schnauzer, Bull Terrier, and English Bulldog.

The study groups were organized and divided into four categories: Insulin-managed patients with DM, non-insulin-managed patients with DM, normoglycemic patients with normal weight, and normoglycemic patients with overweight or obesity. DM was defined as a glycemic level of more than 200 mg/dL according to the American Animal Hospital Association guidelines [15], whereas normoglycemia was defined as a glycemic level of up to 110 mg/dL. Body condition was assessed using a 9-point morphometric body condition index, where patients with a score of 5 of 9 were considered to have an ideal body condition, whereas those with a score of more than 5 were considered overweight, and those with a score of more than 8 are deemed obese, according to the World Small Animal Veterinary Association guidelines [16].

### Samples

Serological analysis was performed on a single sample of peripheral blood obtained by venipuncture of the cephalic vein and arranged in two tubes: One without ethylenediaminetetraacetic acid (EDTA) and one with EDTA. The sample was centrifuged at 5000 rpm for 10 min at 21°C. After plasma and serum were separated, they were subsequently frozen at −80°C until analysis. The samples were taken following 8 h fast without restrictions on water intake. Glycemia samples were stored in sodium fluoride tubes and analyzed on the Mindray BS 480 (VALTEK Diagnostics, Santiago, Chile).

### Autoantibodies

All patients included in this study were assessed for anti-GAD65, anti-IA2, and anti-ZnT8 autoantibodies using the Medizym^®^ anti-GAD Kits and anti-IA-2 and anti-ZnT8 enzyme-linked immunosorbent assay (ELISA) system (Germany). Besides, a 25 μL sample, negative control (CI), positive serum (CII), and calibrators were arranged in a 96-well plate and incubated for 60 min between 19°C and 25°C with shaking at more than 500 rpm, according to the manufacturer’s indications. The plate was read at OD 450 nm and corrected for 620 nm using the NanoQuant Pro TECAN Infinite M200 spectrophotometer (Life Sciences, USA). The anti-GAD65 cutoff value was 5 IU/mL with a sensitivity of 92.3% and specificity of 98.6%. For anti-IA-2, the cutoff value was 10 IU/mL with a sensitivity of 79% and specificity of 83%. In anti-ZnT8, the cutoff value was 10 IU/mL with 99% sensitivity and 68% specificity. Besides, two GAD65-, IA-2-, and Znt8-positive human serum samples were used as positive controls. All samples were analyzed in triplicate, and the intra-assay and inter-assay coefficients of variation for these determinations were all within the ranges specified by the manufacturer (intra-assay of less than 2%; interassay of <4%).

### Statistical analysis

Associations between categorical variables were analyzed using the Chi-square test, considering the association between autoantibodies and the presence of DM. p≤0.05 was used to denote statistical significance. Analysis of variance (ANOVA) was used to evaluate the significance, and differences with p≤0.05 were considered statistically significant.

## Results

Table-1 summarizes the information on the distribution of the autoantibodies according to metabolic conditions (diabetic and non-diabetic) among patients with DM without treatment and with healthy weight or obese nutritional status. One patient in the non-diabetic group was positive for anti-GAD65 (9.21 IU/mL). However, no patients were positive for GAD65 autoantibodies in the group with DM. Using ANOVA, the percentage of patients with DM positive for anti-IA2 was 58.1%, which is significantly different from that of patients found positive for other autoantibodies (p<0.0001), and 9.7% of patients with DM were positive for anti-ZnT8 compared with controls (3.3%). Besides, IA was found in 18/31 patients, Zn was found in 3/31 patients, and GAD was found in 0/31 patients. Of the 18 patients who had anti-IA2, three also had anti-ZnT8.

**Table-1:** Distribution of anti-GAD65, anti-IA2, and anti-ZnT8 autoantibodies in control and diabetic dogs.

Groups	n	Anti-GAD 65		Anti-IA2		Anti-ZnT8		p-value ANOVA
		
		n	%	n	%	n	%	
Treated diabetic	15	0	0.0	9	60.0	0	0.0	<0.0001
Untreated diabetic	16	0	0.0	9	56.3	3	9.7	<0.0001
Normal weight control	15	1	6.7	2	6.7	0	0.0	<0.0001
Overweight control	15	0	0.0	0	0.0	1	3.3	<0.0001

Table showing the distribution of the presence of antibodies in the treated and untreated diabetic groups; and normoglycemic normopese and obese. Differences in the presence of antibodies between groups analyzed by ANOVA with a significant p*<*0.05. GAD65=Glutamic acid decarboxylase, IA2=Islet antigen 2, ZnT8=Zinc transporter isoform 8, ANOVA=Analysis of variance

The association between DM and the presence of autoantibodies had a value of χ^2^=10.01 (p<0.0015). The odds ratio associated with the presence of autoantibodies and DM was 2.99 (p<0.0027), indicating that the presence of positive autoantibodies (mainly IA2) puts dogs in the high-risk zone of developing DM.

## Discussion

This study showed that anti-GAD has a distribution pattern opposite to that reported by Davison [7] and Ahlgren *et al*. [10]. Ahlgren *et al*. [10] have found only one positive autoantibody among 121 patients, whereas Davison *et al*. [7] have found four positive autoantibodies among 30 patients. These results discard this autoantibody as a marker for DM in dogs; human anti-GAD65 tends to remain present in late-age DM [17]. The distribution of autoantibodies against anti-ZnT8 found a positive presence in three patients. In humans, this autoantibody shows a reasonable degree of association with type 1 DM, regardless of the presence of risk or protection HLA haplotypes [18]. Most antibody-positive patients were between 8 and 14 years old, except for two patients aged 3 and 4 years, respectively. In patients positive for antibodies, the lowest glycemic level was 238 mg/dL during analysis.

Finally, our results regarding the distribution of anti-IA2 antibodies showed that over 50% of the patients were positive for this antibody. Only one study has found this autoantibody in dogs: One dog among 30 patients studied [19]. This study contrasts with our study, where anti-IA-2 was the autoantibody more frequently found in our patients. This finding can help assess the age at onset of DM in dogs. Studies on human patients with latent autoimmune DM in adults have shown that this autoantibody tends to decrease in the late onset of DM [20].

Our results should be viewed and applied with caution since we used commercial ELISA kits designed to capture human, rather than canine, autoantibodies. Human proteins differ in sequence from canine proteins for all these antigens, making the test less appropriate for canines; besides, commercial human ELISA plates can be blocked using proteins (e.g., bovine serum albumin) to which canine sera have increased reactivity compared with human sera, giving false-positive results [21].

Similarly, the neutering status was excluded from this study, and due to the small sample size, we included all dogs without differentiation by age at diagnosis. Since autoantibodies tend to peak during diagnosis, knowing how long the dogs in the “treated” group had the disease would be essential, as in human medicine, IA-2 antibodies tend to decrease from the time of diagnosis, whereas GAD tends to remain unchanged [17]. Obesity is relevant when assessing patients with autoimmunity, as in obese human patients with a type 2 DM phenotype; a 9.8% positivity of antibodies against GAD 65 or IA2 has been found. Therefore, it deserves further investigation to determine whether it is a mixed type 1 and type 2 DM [22] or a positivity associated with the inflammatory state of obese patients, as the positivity of these patients to weight loss interventions has not been evaluated [23].

At present, canine DM has not been classified as type 1 DM because of a lack of evidence of the presence of autoantibodies in dogs with DM. This preliminary study has found a high frequency of IA-2 autoantibody positivity using a detection kit for humans, so corroborating this high frequency with a more specific detection system in dogs is necessary. These preliminary results should be taken and applied with caution and corroborated using a canine-specific assay when an ELISA is available for such determination.

## Conclusion

Canine DM has not been classified as type 1 DM because of a lack of evidence of the presence of autoantibodies in dogs with DM. This preliminary study has found a high frequency of IA-2 autoantibody positivity using a detection kit for humans, so corroborating this high frequency with a more specific detection system in dogs is necessary. These preliminary results should be taken and applied with caution and corroborated using a canine-specific assay when an ELISA is available for such detection.

## Authors’ Contributions

FG and FP: Conceived the idea, developed the theory, verified the analytical methods. FG: Carried out the experiment and wrote the manuscript. Both authors discussed the results and contributed to the final manuscript. Both authors read and approved the final manuscript.
